# Epidemiology, etiology, and diagnosis of health care acquired pneumonia including ventilator-associated pneumonia in Nepal

**DOI:** 10.1371/journal.pone.0259634

**Published:** 2021-11-17

**Authors:** Sabina Dongol, Gyan Kayastha, Nhukesh Maharjan, Sarita Pyatha, Rajkumar K. C., Louise Thwaites, Buddha Basnyat, Stephen Baker, Abhilasha Karkey

**Affiliations:** 1 Patan Academy of Health Sciences, Patan Hospital, Oxford University Clinical Research Unit, Kathmandu, Nepal; 2 Patan Academy of Health Sciences, Patan Hospital, Kathmandu, Nepal; 3 The Hospital for Tropical Diseases, Wellcome Trust Major Overseas Programme, Oxford University Clinical Research Unit, Ho Chi Minh City, Vietnam; 4 Cambridge Institute of Therapeutic Immunology & Infectious Disease (CITIID), Department of Medicine, University of Cambridge, Cambridge, United Kingdom; University of Georgia, UNITED STATES

## Abstract

Epidemiologic data regarding health care acquired pneumonia (HAP) and ventilator-associated pneumonia (VAP) from Nepal are negligible. We conducted a prospective observational cohort study in the intensive care unit (ICU) of a major tertiary hospital in Nepal between April 2016 and March 2018, to calculate the incidence of VAP, and to describe clinical variables, microbiological etiology, and outcomes. Four hundred and thirty-eight patients were enrolled in the study. Demographic data, medical history, antimicrobial administration record, chest X-ray, biochemical, microbiological and haematological results, acute physiology and chronic health evaluation II score and the sequential organ failure assessment scores were recorded. Categorical variables were expressed as count and percentage and analyzed using the Fisher’s exact test. Continuous variables were expressed as median and interquartile range and analyzed using Kruskal-Wallis rank sum test and the pairwise Wilcoxon rank—sum test. 46.8% (205/438) of the patients required intubation. Pneumonia was common in both intubated (94.14%; 193/205) and non-intubated (52.36%; 122/233) patients. Pneumonia developed among intubated patients in the ICU had longer days of stay in the ICU (median of 10, IQR 5–15, P< 0.001) when compared to non-intubated patients with pneumonia (median of 4, IQR 3–6, P< 0.001). The incidence rate of VAP was 20% (41/205) and incidence density was 16.45 cases per 1,000ventilator days. Mortality was significantly higher in patients with pneumonia requiring intubation (44.6%, 86/193) than patients with pneumonia not requiring intubation (10.7%, 13/122, p<0.001, Fisher’s exact test). Gram negative bacteria such as *Klebsiella* and *Acinetobacter* species were the dominant organisms from both VAP and non-VAP categories. Multi-drug resistance was highly prevalent in bacterial isolates associated with VAP (90%; 99/110) and non-VAP categories (81.5%; 106/130). HAP including VAP remains to be the most prevalent hospital-acquired infections (HAIs) at Patan hospital. A local study of etiological agents and outcomes of HAP and VAP are required for setting more appropriate guidelines for management of such diseases.

## Introduction

Pneumonia is clinically defined as the presence of a new lung infiltrate with evidence that the infiltrate is triggered by an infectious agent such as, the new onset of fever, purulent sputum, or leukocytosis [1]. Healthcare acquired pneumonia (HAP) is an infection of the pulmonary parenchyma that develops>48 hours of admission to a health care facility and is commonly caused by pathogens that circulate in hospital settings [2]. In clinical practice, HAP is suspected when a patient presents with fever, impaired oxygenation, and suppurative secretions [3]. HAP is an important infectious disease worldwide and is associated with high morbidity, mortality, and additional health system expenditure [4]. In the US, the prevalence of HAP has been estimated to be1.6% of all hospital admissions, representing a rate of 3.63 cases per 1,000 patient-days [5]. Epidemiologic data regarding HAP in Asia are scarce; however, the incidence of HAP is predicted to be high across Asia and especially problematic in intensive care units (ICUs), where the proportion of ICU-acquired respiratory infections ranges from 9% to 23% of admissions [6].

Ventilator-associated pneumonia (VAP) is a subtype of HAP that develops in ICU patients who have been mechanically ventilated for at least 48 hour [2, 7, 8]. VAP remains one of the most common infections in patients requiring invasive mechanical ventilation and is the leading cause of ICU mortality [2, 7]. The reported prevalence of VAP vary from 5 to 40% of ventilated patients depending on country, ICU type, and criteria used to diagnose VAP [9]. In high-income countries, a combination of surveillance, education, and tailored intervention and prevention bundles have led to a major reduction in VAP [10]. However, even with the implementation of such programs, VAP is still commonly reported in the US [7]. In Asia there are limited data on incidence of VAP, the causative pathogens, and their antimicrobial susceptibility profiles [6, 11].A meta-analysis which encompassed 88 studies from 22 Asian countries from 2008 to 2018 indicated that the pooled incidence density of VAP in low-middle-income countries (LMICs) (18.5 per 1,000 ventilator-days) was more than twice that in high-income countries (9.0 per 1,000 ventilator-days) [12].

VAP has received little attention in LMICs until relatively recently [13]. In a low income country, like Nepal, where the incidence of infectious disease is high and strategies for control and prevention are weak, the opportunity for nosocomial infection is significantly higher [11, 14–16].This problem is further exacerbated by antimicrobial resistance (AMR) in organisms such as *Acinetobacter baumannii* and *Klebsiella pneumoniae*, which are responsible for a large proportion of nosocomial infections and commonly multi-drug resistant (MDR) [17, 18].

A delayed diagnosis and delay in initiating appropriate therapy in VAP may be associated with poor outcomes [2, 19–21]. Therefore, an early and accurate diagnosis is fundamental in the management of patients with VAP. In order to develop effective therapeutic strategies to optimize the use of antimicrobial agents we need a better understanding of the local pathogens causing. Therefore, we performed a prospective study to describe some epidemiological features of HAP among patients admitted to the ICU of major tertiary hospital in Kathmandu, Nepal. We measured the incidence rate of VAP, investigated the antimicrobial susceptibility profiles of the etiological agents, and compared clinical profiles associated with HAP/VAP mortality.

## Materials and methods

### Ethics approval and consent to participate

This study was approved by Nepal Health Research Council (NHRC) (Reference number 11/2016,Date: 11 March 2016) and Oxford Tropical Research Ethics Committee (OxTREC 32–16, Date:19 October 2016). Adult patients admitted in the ICU or next-of-kin of the patient were approached for written informed consent to participate in this study.

### Setting and study design

This was a prospective observational cohort study conducted in the ICU of Patan hospital between April 2016 and March 2018. Patan hospital is a 450-bed tertiary care referral teaching hospital with 15 ICU beds, located in the Lalitpur Metropolitan area of the Kathmandu valley in Nepal.

### Study structure

All adult patients, ≥ 18 years of age admitted to the ICU were eligible to participate in the study. Adult patients admitted in the ICU or next-of-kin of the patient were approached for written informed consent to participate. Patients who denied consent and under the 18 years of age were not included in the study. Upon recruitment, demographic data, medical history, antimicrobial administration record, chest X-ray or other imaging findings, biochemical, microbiological and haematological results, and clinical parameters were recorded in a case report form (CRF). The acute physiology and chronic health evaluation (APACHE) II score and the sequential organ failure assessment (SOFA) score were recorded from the biochemical findings of the day of admission. Daily observation of the individual was conducted and CRF completed until an outcome of discharge, death, transfer to another ward or development of VAP.

**Fig 1:**
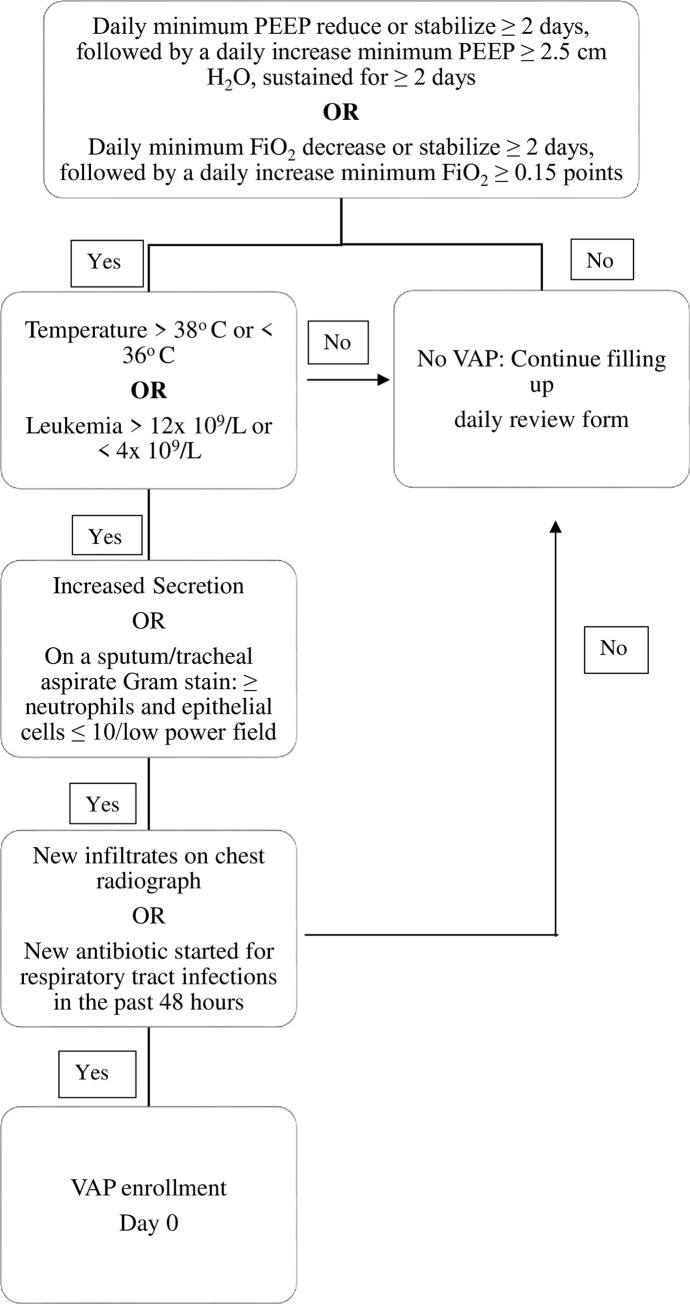
Flow diagram for VAP definition. Firstly, a deterioration in ventilation following a period of stability defined according to positive end expiratory pressure (PEEP): ≥ 2 days of stable or decreasing daily minimum PEEP followed by a rise in daily minimum PEEP of ≥ 2.5 cm H_2_O, sustained ≥ 2 calendar days; or FiO_2_: ≥2 days of stable or decreasing daily minimum fraction of inspired oxygen(FiO_2_) followed by a rise in daily minimum FiO_2_ ≥ 0.15 points, sustained ≥ 2 calendar days. Secondly, systemic signs of fever >38°C or <36°C or white blood cell count >12×10^9^/L or <4×10 ^9^/L were required. Final criteria was an increased/new purulent tracheal aspirate (TA) samples/tracheobronchial secretions or ≥25 neutrophils per low power field (10 objective) on Gram stain of tracheal aspirate and either new and persistent infiltrates, consolidation, or cavitation as read by two study physicians on chest X-ray, or the decision to commence new antibiotic therapy.

VAP was defined by following the modified US Centers for Disease Control and Prevention criteria which requires to fulfill radiographic, systemic, and pulmonary criteria [16, 22–24] “Fig 1”.

The day when the patient fulfilled the criteria of VAP was taken as day 0 of VAP diagnosis. These VAP confirmed patients were followed up on day 3, day 7, and day14. During these visits comparable clinical information was collected via hospital records. The final follow up was conducted on day 30 by phone if the patient was discharged, or in person if the patient was still in the hospital. Final diagnosis or working diagnosis if still under admission were recorded in the patient CRF.

### Sample collection for microbiological culture

Respiratory samples [either tracheal aspirates (TA), bronchoalveolar lavage (BAL), or sputum] and blood samples were obtained from enrolled patients for microbiological culture. A respiratory sample of either TA or BAL was obtained from all patients before the diagnosis of VAP. The decision for BAL or TA samples were at the discretion of the treating physician.

### Collection of TA sample

TA samples were collected as previously described, following local standard operating procedures [25]. Specimens were transported to the microbiology laboratory, and processed within 2 hours of collection. The tracheal aspirate specimens were examined by Gram staining, and the aspirate fluid was diluted 1:1 with Sputasol (Oxoid) and incubated at 37°C, with periodic agitation, until liquefaction. The sample was diluted (1:1, 10^−1^ and 10^−2^) using maximum recovery diluent (Oxoid), and 20 ml 1:1 diluent was inoculated onto blood agar and chocolate agar plates. Additionally, 20μl of the 10^−1^ and 10^−2^ dilutions was plated onto MacConkey media and blood agar base (Mast diagnostics, UK). Inoculated media were incubated at 37°Cand examined after 24 and 48 h of incubation. The threshold used to discriminate between infection and colonization was ≥1x10^5^ colony forming unit (CFU)/ ml^-^1 (i.e., 20 colonies on either media from the 10^−2^ dilution). Colonies above this threshold were identified using biochemical tests following standard operating protocol of Patan Hospital. In the interpretation of results, each colony corresponded to 20,000 CFU/ml, and it was considered to be TA positive when the count was ≥10^5^CFU/ml [26].

### Antimicrobial susceptibility testing

Antimicrobial susceptibility testing was performed using the Kirby Bauer disc diffusion method. The inhibitory zone sizes were interpreted according to the Clinical and Laboratory Standards Institute (CLSI) 2018 guidelines. Mueller–Hinton agar and antimicrobial discs were purchased from Mast Diagnostics, UK. *Escherichia coli* ATCC 25922 and *Staphylococcus aureus* ATCC 25923 were used as controls for these assays. The antimicrobials tested against *Acinetobacter* spp., *Pseudomonas* spp., and the Enterobacteriaceae were amikacin (30 mg), piperacillin/tazobactam (100/10 mg), imipenem (10 mg), ofloxacin (5 mg), and ceftriaxone (30 mg). An isolate was defined as MDR when it was non-susceptible to at least one agent in ≥3 antimicrobial categories[CLSI guidelines (2018)] [27].Gram positive organisms were tested against co-trimoxazole (1.25/ 23.75 mg), penicillin (10 mg), gentamicin (10 mg), erythromycin(15mg) and oxacillin (1 mg).

### Statistical analysis

**Fig 2:**
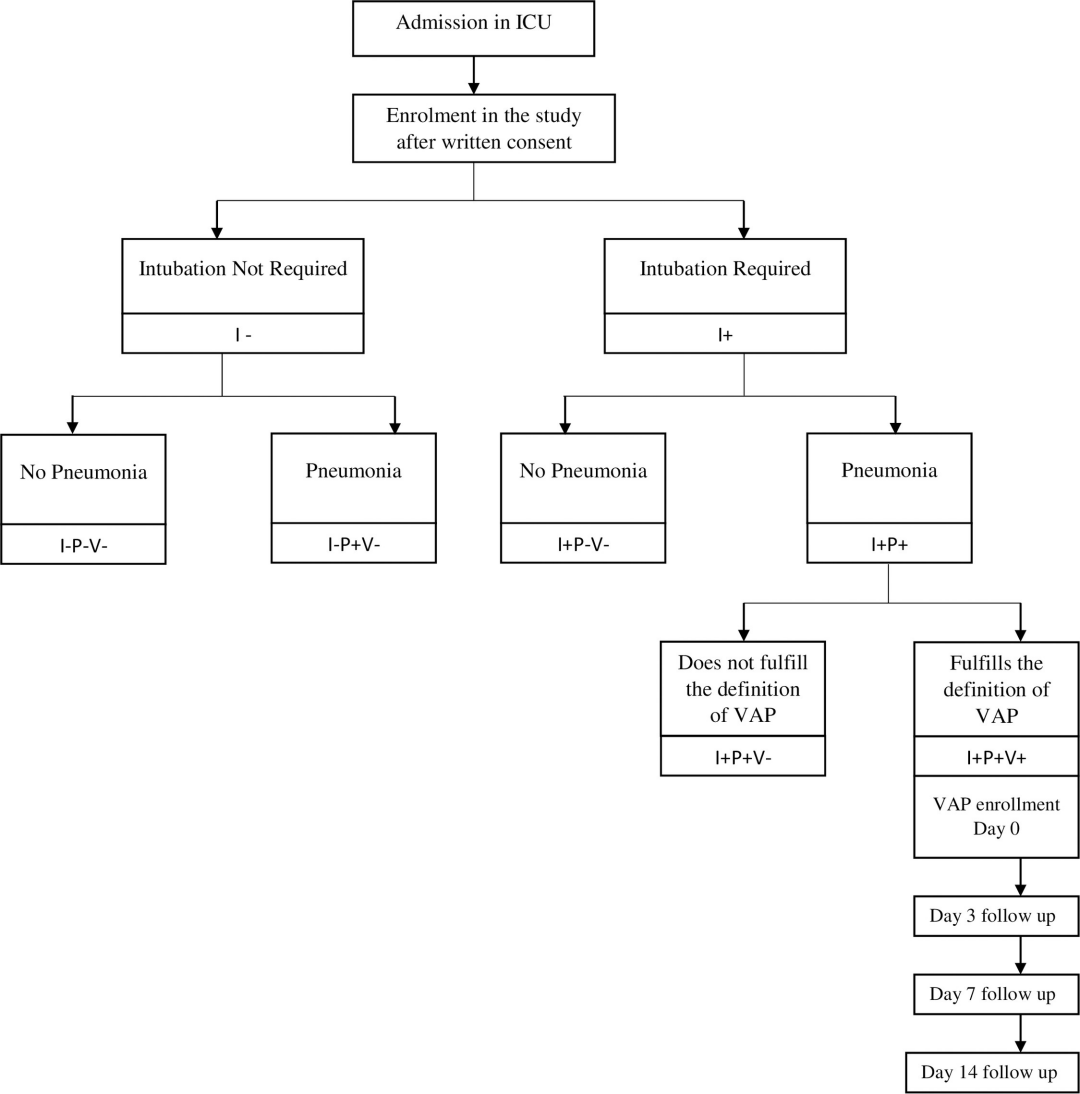
Flow diagram of the study enrollment procedure and categorization into five categories. Patients enrolled in the study were into five groups depending upon intubation, pneumonia development, and VAP development: These were: I-P-V- (not intubated, no pneumonia), I-P+V- (not intubated, but pneumonia developed), I+P-V- (intubated but no pneumonia), I+P+V-(intubated and pneumonia developed, but VAP not confirmed), and I+P+V+(VAP confirmed). The flow diagram of the study enrollment procedure and categorization into five categories is shown in “Fig 2”.

Data recorded onto a case record form were entered into a CliRes database system protecting participant information. Verification was done by double entry. Data analysis was performed in R Software (version 3.2). Categorical variables were expressed as count and percentage and analyzed using the Fisher’s exact test. Continuous variables were expressed as median and interquartile range and analyzed using Kruskal-Wallis rank sum test and the pairwise Wilcoxon rank—sum test. Each variable with a p- value < 0.05 was considered a significant variable. VAP incidence was calculated as follows: (Number of cases with VAP/Total number of patients who received MVx100) = VAP rate per 100 patients. VAP incidence density was calculated as follows: (Number of cases with VAP/Number of ventilator days) x 1000 = VAP per 1,000 ventilator days. Flow diagram of the study enrollment procedure and categorization into five categories is shown in “Fig 2”.

## Results

### Baseline characteristics

Four hundred and thirty-eight patients between April 2016 and March 2018were hospitalized in the ICU and enrolled in the study. The patients were between the ages of 18 and 95 years and 48.9% (214) were male and 51.1% (224) were female “Table 1”.

**Table 1:** Baseline characteristics of patients enrolled in the study.

Variables	I-P-V- (111)	I-P+V- (122)	I+P-V- (12)	I+P+V- (152)	I+P+V+ (41)
Age (years)	48 (30–62)	62.5 (48.5–72)	35.5 (23.3–50.3)	57.5 (35–72)	59.5 (42.8–76.3)
Weight (Kilogram)	59.5 (50–65)	57 (49.5–64)	60 (50.5–70.5)	58 (50–65)	58 (49–62)
M:F	57:54	53:69	7:5	77:75	20:21
SOFA	3 (2–5)	3 (2–5)	6.5 (4–10)	8 (5–11)	10 (7–11)
APACHE	10 (6–14)	11 (7.3–14)	12 (6.8–15.3)	18 (13–24)	17 (13–22)
Comorbidity	60 (54.1%)	90 (73.8%)	7 (58.3%)	91 (59.9%)	27 (65.9%)
Hospital previous 90 days	22 (19.8%)	19 (15.6%)	3 (25%)	38 (25%)	7 (17.1%)
Antibiotics in last 90 days	11 (9.9%)	28 (23%)	3 (25%)	54 (35.5%)	4 (9.8%)

Values given are median (IQR) or count (percent).

I-P-V- (Non intubated, no pneumonia), I-P+V- (non- intubated, pneumonia developed), I+P-V- (intubated, no pneumonia), I+P+V- (intubated, pneumonia but not VAP also called non–VAP), I+P+V+ (intubated, pneumonia and VAP confirmed).

The total numbers of participants in each of the five categories were I-P-V- = 111, I-P+V- = 122, I+P-V- = 12, I+P+V- = 152, and I+P+V+ = 41, who had median ages of 48, 62.5, 35.5, 57.5, and 59.5years, respectively. More than 50% of participants in all five categories had co-morbidities at the time of admission in the ICU with >15% of the participants in each category having had a history of hospital admission in the past 90 days with antimicrobial use ranging from 9.8% (4/41 in the I+P+V+ group) to 35.5% (54/152 in the I+P+V- group). The median SOFA scores ranged from 3 to 10, with the highest scores being observed in the VAP group (median of 10, IQR 7–11). The median APACHE II score ranged from 10 to 18; the highest score was observed in the non-VAP group 18 (IQR 13–24).

### Association of different variables with intubation and pneumonia

In total, 29.7% (130/438) of patients had a diagnosis of pneumonia when admitted in the ICU and 46.8% (205/438) of patients required intubation. The most common requirement for intubation was failure to oxygenate (24.4%; 50/205), followed by failure to maintain or protect the airway (99%; 39/205). Pneumonia was common in both the intubated (94.14%; 193/205) and the non-intubated (52.36%; 122/233) patients. Pneumonia in intubated patients in the ICU was associated with longer days of stay (median of 10, IQR 5–15, *p*< 0.001) when compared to non-intubated patients with pneumonia (median of 4, IQR 3–6, *p*< 0.001). SOFA and APACHE II scores were also significantly higher among the patients that then on-intubated patients (*p*<0.05; Kruscal-wallis rank sum test) “Table 2”.

**Table 2:** Kruskal-wallis rank sum test for the variables associated with intubation and pneumonia.

Variables	IntPosPneum	NonIntPosPneum	p—value
	I+P+V(V+and V-)	I-P+V-	
	(193)	(122)	
Days in Hospital	11 (7–17)	5 (3–7)	<0.001
Days in ICU	10 (5–15)	4 (3–6)	<0.001
Days in Intubation	9 (5–15)	NA	NA
FiO2	50 (40–80)	36 (29–41)	<0.001
PaO2	79 (48.2–114)	68.1 (53.7–88)	0.056
Temperature	98 (97.2–99.2)	98 (97.2–98.4)	0.027
SOFA	8 (5–11)	3 (2–5)	<0.001
APACHE	18 (13–23)	11 (7.25–14)	<0.001
Mortality	86/193 (44.6%)	13/122 (10.7%)	<0.001

Values given are median (IQR) or count (percent). Kruskal-Wallis rank sum test for median (IQR). Fisher’s Exact Test for count (percent). I+P+ (intubated, pneumonia) and I-P+ (non- intubated, pneumonia developed).

### Incidence density of VAP

Out of 205 patients requiring mechanical ventilation during their stay in ICU, 41 patients were diagnosed with VAP (20%), equating with a total incidence density of 16.45 cases per 1,000 ventilator days.

### Factors associated with VAP confirmed cases

We aimed to identify factors associated with VAP in the VAP confirmed group (I+P+V+) “Table 3”. VAP was significantly associated with the duration of stay in the hospital (median 18 days, IQR 11–27, *p*<0.001), the duration of stay in ICU (median16 days, IQR 10–25, *p*<0.001), number of days of intubation (median 17 days, IQR 11–27, *p*<0.001), fraction of inspired oxygen(FiO2) (median 60, IQR 40–100, *p*<0.001), APACHE II score (median 17, IQR 13–22 *p*<0.001), SOFA score (median 10, IQR 7–11, *p*<0.001) and PaO_2_ (median 70.6, IQR 52.3–102, *p*<0.04).

**Table 3:** Kruskal-Wallis rank sum test for the variables associated with different categories.

	I-P-V- (n = 111)	I-P+V- (n = 122)	I+P-V- (n = 12)	I+P+V- (n = 152)	I+P+V+ (n = 41)	p—value
Days in Hospital	4 (3–6)	5 (3–7)	5 (2.75–7)	10 (7–14)	18 (11–27)	<0.001
Days in ICU	3 (2–6)	4 (3–6)	4.5 (2.75–6)	9 (5–12)	16 (10–25)	<0.001
Days in Intubation	NA	NA	4 (2.75–7.25)	7.5 (4–11)	17 (11–27)	<0.001
FiO2	29 (21–34)	36 (29–41)	40 (30–41)	50 (40–70)	60 (40–100)	<0.001
PaO2	85 (65.8–111.4)	68.1 (53.7–88)	103 (64–150.5)	82.5 (48.1–119)	70.6 (52.3–102)	0.041
Temperature	98 (97–98.7)	98 (97.2–98.4)	98.2 (97–99.2)	98 (97.1–99.15)	98.6 (98–99.2)	0.085
SOFA	3 (2–5)	3 (2–5)	6.5 (4–10)	8 (5–11)	10 (7–11)	<0.001
APACHE	10 (6–14)	11 (7.25–14)	12 (6.75–15.25)	18 (13–24)	17 (13–22)	<0.001
Mortality	4/109(3.7%)	13/122(10.7%)	3/12 (25%)	62/155 (40%)	24/41 (58.5%)	<0.001

Values given are median (IQR) or count (percent). Kruskal-Wallis rank sum test for median (IQR).

Fisher’s Exact Test for count (percent).

**Fig 3:**
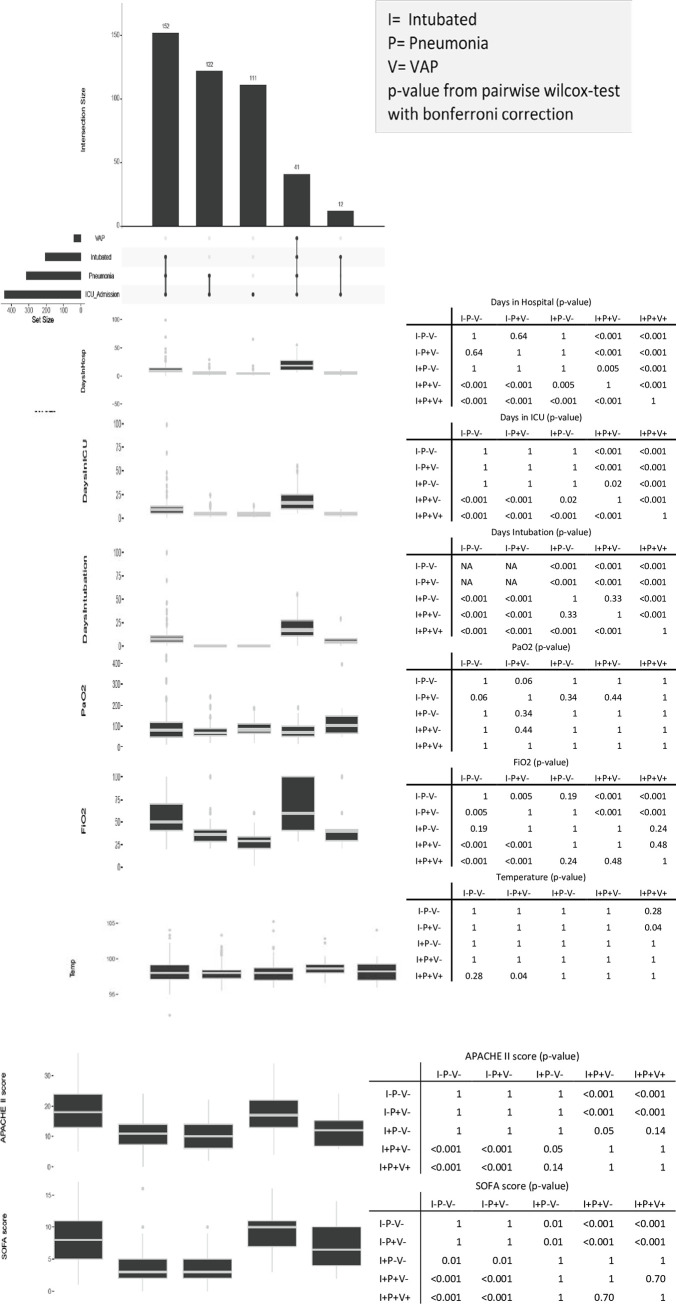
Pairwise Wilcoxon signed rank test performed among the five defined categories to identify significance between the groups. I = intubated, P = pneumonia, V = VAP. P-value from pairwise wilcox-test with bonferroni correction.

A pairwise Wilcoxon signed rank test was performed among the five defined categories to identify significance between the groups “Fig 3”. The number of days of hospital stay, the number of days of ICU stay, APACHE II score, and SOFA score were all significantly higher in the I+P+V+ and I+P+V- groups than in the I-P-V- group (*p*< 0.001).

### Mortality

Mortality was significantly higher in patients with pneumonia requiring intubation than patients with pneumonia not requiring intubation (44.6% (86/193) vs. 10.7%, (13/122)*p*<0.001, Fisher’s exact test) “Table 2”. Between the groups, the highest mortality (58.5%; 24/41) was observed among VAP patients (I+P+V+) followed by non-VAP (I+P+V-) patients (40.8%,62/152); and lowest mortality (3.7%; 4/109) was observed among patients who neither required intubation nor had pneumonia during their stay in the ICU (I-P-V-)(*p*< 0.001; Kruskal wallis test) “Table 3”.

### Microbiology of VAP and non- VAP

A total of 110 samples from those with confirmed VAP(I+P+V+) and 130 samples from those without-VAP category (includes all categories except I+P+V+) were subjected to microbiological cultured. The majority of these samples were TA samples; 81/110 in VAP category and 63/130 in non-VAP category.

**Fig 4:**
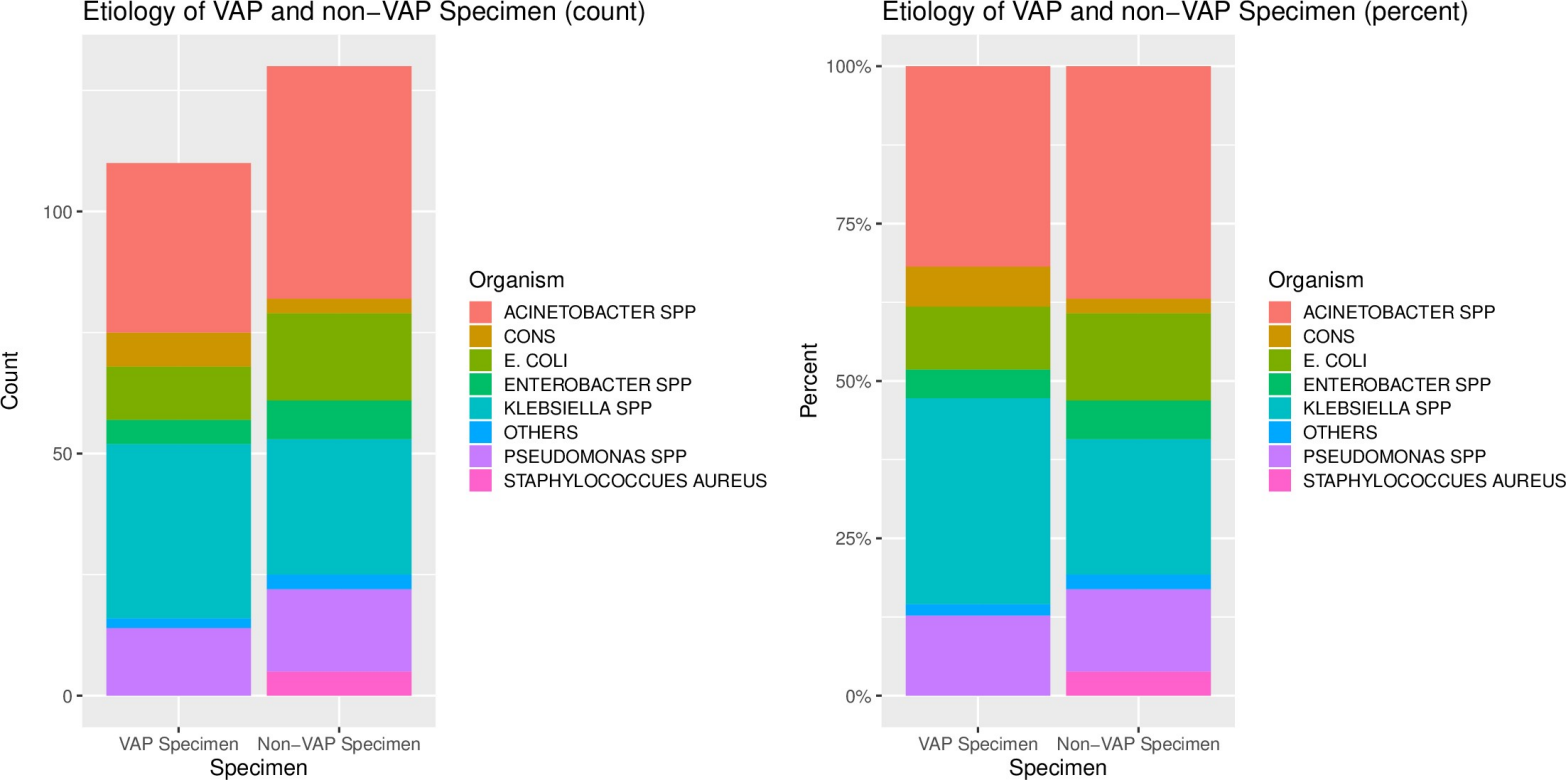
Etiology of VAP and non-VAP specimen.

Gram negative bacteria were the dominant organisms from both VAP and non-VAP patients “Table 4”, “Fig 4”). *Klebsiella* species was the most common bacteria associated with VAP (n = 36,32.7%) followed by *Acinetobacter* species (n = 35, 31.8%).*Acinetobacter* species was the predominant organism (n = 48, 36.9%) isolated from those without-VAP, followed by *Klebsiella* species (n = 28, 21.5%).*Klebsiella* species was more likely to be isolated from VAP patients (OR1.76, 95%CI 0.96–3.3,p value 0.05).

**Table 4:** Etiology of VAP and non-VAP specimens.

Isolates	VAP (n = 110)	Non-VAP (n = 130)	OR (95%CI)	p-value
*Acinetobacter* spp	35 (31.8%)	48 (36.9%)	0.8 (0.45–1.4)	0.42
*Klebsiella* spp	36 (32.7%)	28 (21.5%)	**1.76 (0.96–3.3)**	**0.05**
*Pseudomonas* spp	14 (12.7%)	17 (13.1%)	0.97 (0.42–2.21)	1
*E*. *coli*	11 (10%)	18 (13.8%)	0.69 (0.28–1.64)	0.43
*Enterobacter* spp	5 (4.5%)	8 (6.2%)	0.73 (0.18–2.61)	0.78
Coagulase negative *Staphylococcus* (CoNS)	7 (6.4%)	3 (2.3%)	2.9 (0.6–17.6)	0.19
*S*. *aureus*	0 (0%)	5 (3.8%)	NA	NA

### MDR in VAP and non-VAP categories

**Fig 5:**
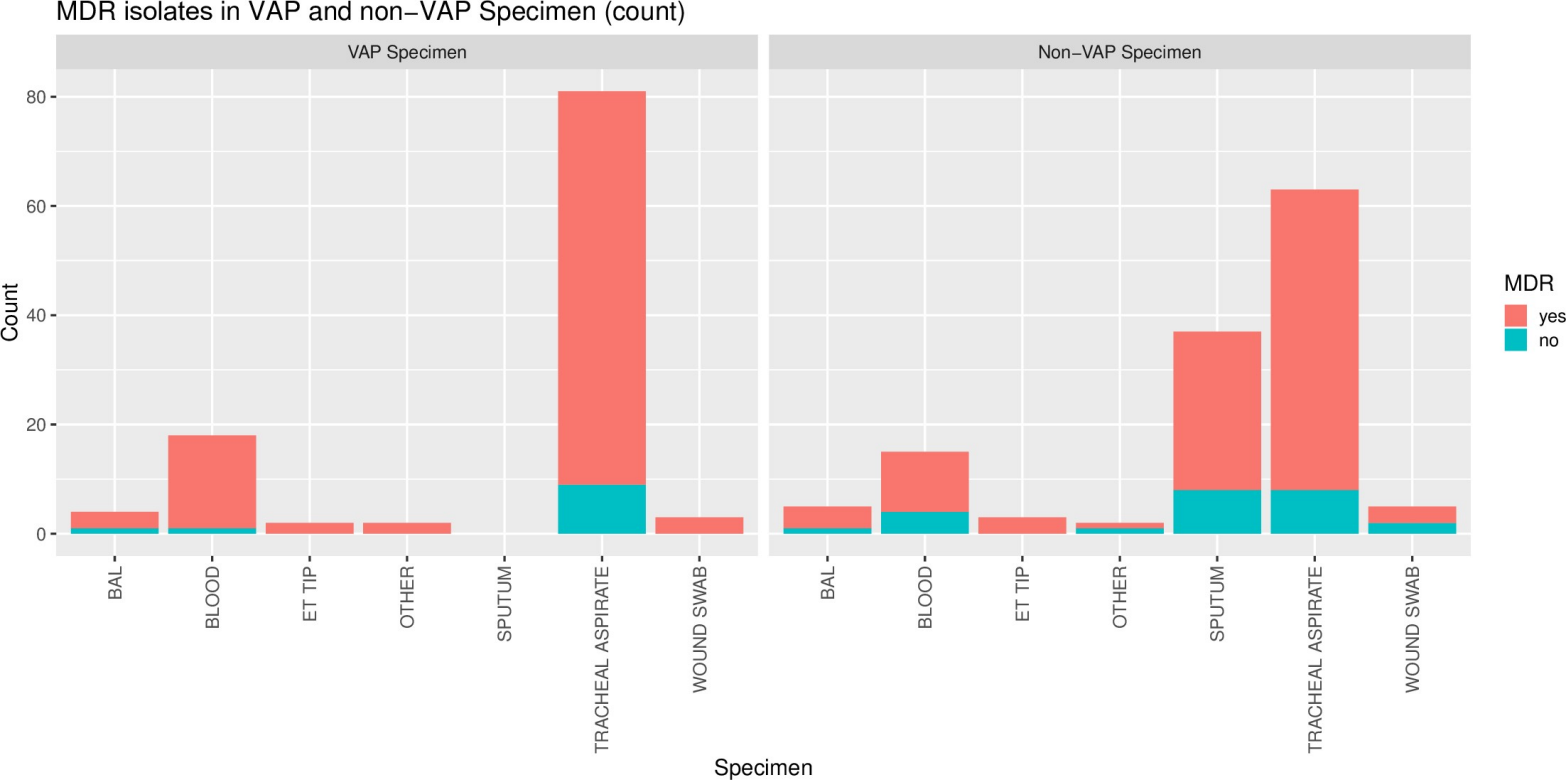
MDR isolates in VAP and non-VAP specimen.

MDR was prevalent in all bacterial isolates from both VAP and non-VAP categories. The distribution of MDR isolates from various VAP and non-VAP samples are presented in “Fig 5”. 90% (n = 99/110) of the isolates from various VAP samples and 81.5% (n = 106/130) of the non-VAP isolates were MDR. The data was suggestive of association of MDR with the VAP isolates but this was none significant (OR 2.03, 95%CI 0.90–4.85, p 0.07).

## Discussion

The data on HAP from prospective studies are scant notably from LMIC setting. Our study showed that HAP was common in our ICU setting regardless of intubation requirement [94.14% (193/205) among intubated and 52.36% (122/233) among non-intubated patients] indicating that these pneumonia cases may be a common HAI at Patan hospital. In addition to this, at least 16% of the patients from all the categories have had a visit to the hospital in the past 90 days and 50% of the patients had co-morbidities “Table 1” mainly chronic respiratory illness such as chronic obstructive pulmonary disorder. As a result, antibiotic usage was also common. Pneumonia as HAP was also common in a Malaysian study where,21% of HAP infections were pneumonia [6].

The complex interplay between the endotracheal tube, presence of risk factors, virulence of the invading bacteria and host immunity largely determine the development of VAP [28].The diagnosis of VAP is traditionally based on clinical symptoms and radiographic criteria that require further bacteriological confirmation. However, it has been demonstrated that these criteria are not sensitive or specific [8]. There is no gold standard for the diagnosis of VAP however, the qualitative method of culturing the tracheobronchial aspirate samples is said to be better at differentiating colonization and actual infection.

Despite recent advances in microbiological tools, the epidemiology and diagnostic criteria for VAP are still controversial, complicating the interpretation of treatment, prevention, and outcomes studies [9]. Data on rates of VAP, the common associated pathogens, and their antimicrobial susceptibility profiles from Asia are limited [12]. Pooled incidence density of VAP was 18.5 per 1000 ventilator days in high in Asian LMIC countries [12]. This finding is similar to our study where we observed the incidence rate of VAP of 20% (n = 41/205) with a total of incidence density of 16.45 per 1000 ventilator days. However, some other Asian countries have reported a lower VAP incidence (9.9%) and VAP density (8.7/1000 ventilator days) [16]. Meta- analysis study from mainland China reported that the cumulative incidence of VAP was 23.8% [29]. In contrast, studies from India have reported the higher incidence density of VAP of 39.6% to 40.1% [30, 31].Such reported incidences vary widely from 5 to 40% depending on the setting and diagnostic criteria [9] indicating that the incidence rates vary not only between countries but also among different settings within a country. In high-income countries, a combination of surveillance, education, and tailored intervention and prevention bundles have led to a reduction in the incidence of VAP [32].

Gram negative bacteria were the dominant organisms from both VAP and non-VAP categories. *Klebsiella* species was the most common bacteria associated with VAP followed by *Acinetobacter* species Among non-VAP category, *Acinetobacter* species was the predominant organism followed by *Klebsiella* species (n = 28, 21.5%). *Klebsiella* species was more likely to be isolated from VAP category). We did not find major differences in the etiologic agents of VAP and non-VAP organism and their antimicrobial susceptibility profiles. However, there was a suggestive association of MDR with VAP isolates. Similar findings have been reported from other studies where the leading pathogens are *A*. *baumannii*, *P*. *aeruginosa* and *K*. *pneumoniae* [33, 34]. In a large meta-analysis of 88 studies analyzing VAP in adults in Asia, it was revealed that *A*. *baumannii* was the most common organism in the LMIC group and the proportion due to this organism gradually reduced as income levels increased, and *S*. *aureus* and *P*. *aeruginosa* were the most common in the high income country group [12].Studies on VAP from other Asian countries also have reported *A*. *baumannii* to be the most common isolate [35].

One of the differences between our data and reports from Western countries was the proportion of gram- negative and gram-positive bacterial causes of VAP. We found a much lower proportion of gram-positive organisms as a causative agents of VAP and non VAP [35, 36].

ICUs often have the highest levels of infections due to antimicrobial resistant pathogens as a result of the environment that is under constant pressure with high antimicrobial usage due to the presence of severely ill patients. Etiologic agents of VAP are generally associated with pathogens with high levels of antimicrobial resistance, resulting in the need to treat with broad-spectrum antibiotics, which further drives antibiotic resistance [12].

Early onset VAP is usually attributed to antibiotic sensitive pathogens whereas late onset VAP is more likely caused by MDR bacteria and emerges after 4 days of intubation [37, 38]. However, this scenario seems to be different in the LMIC settings. This study revealed that MDR isolates were slightly higher in VAP than in non-VAP categories. Although significant association was not observed, there was an indication of association of VAP with MDR organisms. This further highlights the need to have infection control protocol guidelines in order to control such HAIs. s Guidelines for VAP prevention, including hand washing, elevation of the head of the bed, oral care with chlorhexidine, optimized endotracheal tube cuff pressure, respiratory circuit manipulation, and weaning protocols to early extubation were established in our hospital. These are cost effective control and preventive measures of VAP. Strict compliance, staff training, and regular monitoring of implementation of such guidelines will be effective in the prevention of VAP.

Mortality attributable to HAP is estimated between 5 and 13% [39].Even in HAP, generally considered to be less severe than VAP, serious complications occur in approximately 50% of patients [40].Mortality was significantly higher in patients with pneumonia requiring intubation (44.6%, 86/193) than patients with pneumonia without intubation (10.7%, 13/122, p< 0.001). Highest mortality of 58.5% (24/41) was observed among VAP patients (I+P+V+) followed by non VAP (I+P+V-) patients (40.8%,62/152) and lowest mortality of 3.7% (4/109) was observed among patients who neither required intubation nor had pneumonia during their stay in the ICU (I-P-V-) category. The mortality in our ICU due to VAP was still lower than reported in other studies where it was as high as 68.4% [31].

Development of pneumonia increased the number of days of intubation. Intubated patients with pneumonia had to spend a median of 7.5(4–11) (I+P+V-) to 17(11–27) (I+P+V+) days being intubated as in comparison to those without pneumonia (I+P-V-) {4(2.75 to 7.25)}. In addition, VAP confirmed patients spent a median of16 days in ICU ranging from 10 to 27 days.24.6% (50 / 203) patients required intubation due to the reduction in exchange of oxygen (low PaO2), followed by cognitive impairment and airway obstruction (19.2%, 39/203).

## Conclusion

Pneumonia was one of the common infections in our ICU setting. Pneumonia developed among intubated patients in the ICU had longer days of stay in the ICU when compared to non-intubated patients with pneumonia. We found high VAP incidence in this study and highest mortality was observed among VAP patients followed by non VAP (I+P+V-) patients. MDR Gram negative bacteria were the dominant organisms from both VAP and non-VAP categories.

HAP including VAP remains to be the most prevalent HAIs at Patan hospital. One of the limitations of this study was that it was conducted at a single hospital. Surveillance studies on HAIs at various hospitals within the country are required in identifying the etiological agents. Antimicrobial susceptibility profiles of the etiological agents and outcomes of HAP and VAP would be beneficial for setting more appropriate guidelines for management of such diseases. In addition, countries like Nepal lack proper protocols of infection control and implementation for minimizing such infections in the hospital. Therefore, a suitable surveillance programs should be implemented, analyzing differences in VAP rates between different ICUs, and evaluating potential therapeutic approaches, and prevention strategies.

## Supporting information

S1 Dataset (XLSX)

## References

[1] Ewig S, Bauer T, Torres A. The pulmonary physician in critical care · 4: Nosocomial pneumonia. *Thorax* 2002; 57: 366–371. doi: 10.1136/thorax.57.4.366 11923560 PMC1746297

[2] Thoracic American, Society H. Guidelines for the Management of Adults with Hospital-acquired, … *Am J Respir Crit Care Med*. Epub ahead of print 2005. doi: 10.1164/rccm.200405-644ST 15699079

[3] Leone M, Bouadma L, Bouhemad B, et al. Hospital-acquired pneumonia in ICU. *Anaesth Crit Care Pain Med* 2018; 37: 83–98. doi: 10.1016/j.accpm.2017.11.006 29155054

[4] Welte T, Torres A, Nathwani D. Clinical and economic burden of community-acquired pneumonia among adults in Europe. Thorax 2012; 67: 71–79. doi: 10.1136/thx.2009.129502 20729232

[5] Giuliano KK, Baker D, Quinn B. The epidemiology of nonventilator hospital-acquired pneumonia in the United States. *Am J Infect Control* 2018; 46: 322–327. doi: 10.1016/j.ajic.2017.09.005 29050905

[6] Chawla R. Epidemiology, etiology, and diagnosis of hospital-acquired pneumonia and ventilator-associated pneumonia in Asian countries. *Am J Infect Control*; 36. Epub ahead of print 2008. doi: 10.1016/j.ajic.2007.05.011 18468551

[7] Chastre J, Fagon J. State of the Art Ventilator-associated Pneumonia. *Am J Respir Crit Care Med* 2002; 165: 867–903. doi: 10.1164/ajrccm.165.7.2105078 11934711

[8] Timsit JF, Esaied W, Neuville M, et al. Update on ventilator-associated pneumonia. F1000Research 2017; 6: 1–13. doi: 10.12688/f1000research.12222.1 29225790 PMC5710313

[9] Papazian L, Klompas M, Luyt CE. Ventilator-associated pneumonia in adults: a narrative review. *Intensive Care Med* 2020; 46: 888–906. doi: 10.1007/s00134-020-05980-0 32157357 PMC7095206

[10] Torres A, Niederman MS, Chastre J, et al. Summary of the international clinical guidelines for the management of hospital-acquired and ventilator-acquired pneumonia. *ERJ Open Res* 2018; 4: 00028–02018. doi: 10.1183/23120541.00028-2018 29977898 PMC6018155

[11] Ranjit S, Bhattarai B. Incidence and risk factors for ventilator-associated pneumonia in Kathmandu University Hospital. *Kathmandu Univ Med J* 2011; 9: 28–31. doi: 10.3126/kumj.v9i1.6258 22610805

[12] Bonell A, Azarrafiy R, Huong VTL, et al. A Systematic Review and Meta-analysis of Ventilator-associated Pneumonia in Adults in Asia: An Analysis of National Income Level on Incidence and Etiology. *Clin Infect Dis* 2019; 68: 511–518. doi: 10.1093/cid/ciy543 29982303 PMC6336913

[13] Resar R, Pronovost P, Haraden C, et al. Using a bundle approach to improve ventilator care processes and reduce ventilator-associated pneumonia. *Jt Comm J Qual Patient Saf* 2005; 31: 243–248. doi: 10.1016/s1553-7250(05)31031-2 15960014

[14] Rosenthal VD, Rodríguez-Calderón ME, Rodríguez-Ferrer M, Singhal T, Pawar M, Sobreyra-Oropeza M, et al. Findings of the International Nosocomial Infection Control Consortium (INICC), Part II: Impact of a multidimensional strategy to reduce ventilator-associated pneumonia in neonatal intensive care units in 10 developing countries. *Infect Control Hosp Epidemiol* 2012; 33: 704–10. doi: 10.1086/666342 22669232

[15] Ohara H, Pokhrel BM, Dahal RK, et al. Fact-finding Survey of Nosocomial Infection Control in Hospitals in Kathmandu, Nepal-A Basis for Improvement. *Trop Med Health* 2013; 41: 113–9. doi: 10.2149/tmh.2013-03 24155652 PMC3798410

[16] Phu VD, Nadjm B, Duy NHA, et al. Ventilator-associated respiratory infection in a resource-restricted setting: Impact and etiology. *J Intensive Care* 2017; 5: 1–9. doi: 10.1186/s40560-016-0195-7 29276607 PMC5738227

[17] Pontikis K, Karaiskos I, Bastani S, et al. Outcomes of critically ill intensive care unit patients treated with fosfomycin for infections due to pandrug-resistant and extensively drug-resistant carbapenemase-producing Gram-negative bacteria. *Int J Antimicrob Agents* 2014; 43: 52–9. doi: 10.1016/j.ijantimicag.2013.09.010 24183799

[18] Leblebicioglu H, Rosenthal VD, Arikan O a, et al. Device-associated hospital-acquired infection rates in Turkish intensive care units. Findings of the International Nosocomial Infection Control Consortium (INICC). *J Hosp Infect* 2007; 65: 251–257. doi: 10.1016/j.jhin.2006.10.012 17257710

[19] Joshi SG, Litake Suresh G Joshi GM, Litake GM, et al. Acinetobacter baumannii: An emerging pathogenic threat to public health. Epub ahead of print 2013. doi: 10.5495/wjcid.v3.i3.25

[20] Luna CM1, Vujacich P, Niederman MS, Vay C, Gherardi C, Matera J JE. Impact of BAL data on the therapy and outcome of ventilator-associated pneumonia. *Chest* 1997; 111: 676–85. doi: 10.1378/chest.111.3.676 9118708

[21] Iregui M, Ward S, Sherman G, et al. Clinical Importance of Delays in the Initiation of Appropriate Antibiotic Treatment for Clinical Importance of Delays in the Initiation of Appropriate Antibiotic Treatment for Ventilator-Associated Pneumonia *. Epub ahead of print 2007. doi: 10.1378/chest.122.1.262 10.1378/chest.122.1.262 12114368

[22] Klompas M, Kleinman K, Khan Y, et al. Rapid and reproducible surveillance for ventilator-associated pneumonia. *Clin Infect Dis* 2012; 54: 370–377. doi: 10.1093/cid/cir832 22247300

[23] Johanson WG, Pierce AK, Sanford JP, et al. Nosocomial respiratory infections with gram-negative bacilli. The significance of colonization of the respiratory tract. *Ann Intern Med* 1972; 77: 701–706. doi: 10.7326/0003-4819-77-5-701 5081492

[24] Centers for Disease Control and Prevention. Pneumonia (Ventilator-associated [VAP] and non-ventilator-associated Pneumonia [PNEU]) Event. *Centers Dis Control Prev* 2015; 2015: 1–15.

[25] Baker S, Thieu Nga TV, Thi Loan H, et al. In vitro activity of colistin in antimicrobial combination against carbapenem-resistant Acinetobacter baumannii isolated from patients with ventilator-associated pneumonia in Vietnam. *J Med Microbiol* 2015; 64: 1162–1169. doi: 10.1099/jmm.0.000137 26297024 PMC4755130

[26] Frota OP, Ferreira AM, Da Silva Barcelos L, et al. Collection of tracheal aspirate: Safety and microbiological concordance between two techniques. *Rev da Esc Enferm* 2014; 48: 618–624. doi: 10.1590/s0080-623420140000400007 25338241

[27] Magiorakos AP, Srinivasan A, Carey RB, et al. Multidrug-resistant, extensively drug-resistant and pandrug-resistant bacteria: An international expert proposal for interim standard definitions for acquired resistance. *Clin Microbiol Infect* 2012; 18: 268–281. doi: 10.1111/j.1469-0691.2011.03570.x 21793988

[28] Kalanuria AA, Zai W, Mirski M. Introduction. *Crit Care* 2014; 18: 208. doi: 10.1186/cc13775 25029020 PMC4056625

[29] Xu Y, Lai C, Xu G, et al. Risk factors of ventilator-associated pneumonia in elderly patients receiving mechanical ventilation. *Clin Interv Aging* 2019; 14: 1027–1038. doi: 10.2147/CIA.S197146 31289438 PMC6566835

[30] Patil H V., Patil VC. Incidence, bacteriology, and clinical outcome of ventilator-associated pneumonia at tertiary care hospital. *J Nat Sci Biol Med* 2017; 8: 46–55. doi: 10.4103/0976-9668.198360 28250674 PMC5320823

[31] Mathai A, Phillips A, Isaac R. Ventilator-associated pneumonia: A persistent healthcare problem in Indian Intensive Care Units! *Lung India* 2016; 33: 512–516. doi: 10.4103/0970-2113.188971 27625445 PMC5006331

[32] Torres A, Niederman MS, Chastre J, et al. International ERS/ESICM/ESCMID/ALAT guidelines for the management of hospital-acquired pneumonia and ventilator-associated pneumonia. *Eur Respir J*; 50. Epub ahead of print 2017. doi: 10.1183/13993003.00582–2017 28890434

[33] Feng DY, Zhou YQ, Zou XL, et al. Differences in microbial etiology between hospital-acquired pneumonia and ventilator-associated pneumonia: A single-center retrospective study in guang zhou. *Infect Drug Resist* 2019; 12: 993–1000. doi: 10.2147/IDR.S204671 31118705 PMC6503191

[34] Werarak P, Kiratisin P, Thamlikitkul V. Hospital-acquired pneumonia and ventilator-associated pneumonia in adults at Siriraj Hospital: Etiology, clinical outcomes, and impact of antimicrobial resistance. *J Med Assoc Thail*; 93. 20364567

[35] Inchai J, Pothirat C, Liwsrisakun C, et al. Ventilator-associated pneumonia: Epidemiology and prognostic indicators of 30-day mortality. *Jpn J Infect Dis* 2015; 68: 181–186. doi: 10.7883/yoken.JJID.2014.282 25672347

[36] Vincent J, Marshall J, Anzueto A, et al. and Outcomes of Infection in Intensive Care Units. *Jama* 2009; 302: 2323–2329. doi: 10.1001/jama.2009.1754 19952319

[37] Kalanuria AA, Zai W, Mirski M. Ventilator-associated pneumonia in the ICU. *Crit Care* 2014; 18: 1–8. doi: 10.1186/cc13775 25029020 PMC4056625

[38] Pneumonia H. Guidelines for the management of adults with hospital-acquired, ventilator-associated, and healthcare-associated pneumonia. *Am J Respir Crit Care Med* 2005; 171: 388–416. doi: 10.1164/rccm.200405-644ST 15699079

[39] Melsen WG, Rovers MM, Groenwold RHH, et al. Attributable mortality of ventilator-associated pneumonia: A meta-analysis of individual patient data from randomised prevention studies. *Lancet Infect Dis* 2013; 13: 665–671. doi: 10.1016/S1473-3099(13)70081-1 23622939

[40] Kalil AC, Metersky ML, Klompas M, et al. Management of Adults With Hospital-acquired and Ventilator-associated Pneumonia: 2016 Clinical Practice Guidelines by the Infectious Diseases Society of America and the American Thoracic Society. *Clin Infect Dis* 2016; 63: e61–e111. doi: 10.1093/cid/ciw353 27418577 PMC4981759

