# Supplementary material for: Epidemiology, etiology, and diagnosis of health care acquired pneumonia including ventilator-associated pneumonia in Nepal
Source: PLoS One. 2021 Nov 17;16(11):e0259634. doi: 10.1371/journal.pone.0259634 (PMC8598067; doi:10.1371/journal.pone.0259634)
Supplement: Attachment — Submitted filename: vap.docx [file pone.0259634.s002.docx]

Reviewer Comment For Editor/Editor-in-Chief

In this manuscript, the authors describe the results of “Epidemiology, etiology, and diagnosis of health care acquired pneumonia including ventilator-associated pneumonia in Nepal”. The results showed that the Multi-drug resistance was highly prevalent in bacterial isolates associated with VAP (90%; 99/110) and non-VAP categories (81.5%; 106/130). HAP including VAP remains to be the most prevalent HAIs at Patan hospital.. However, this article has several drawbacks that are mentioned below.

All abbreviations in the text must be spelled correctly (first in full).

English grammar needs to be revised.

CFU / ml must be spelled correctly in the text.

The sample was collected between 2016-2018, but the methods used CLSI 2016, this is a problem.

What is the meaning of CONS(coagulase-negative staphylococci)? Please refer to it in Table 5.

In the first paragraph of the discussion, it is better to first explain about health care acquired pneumonia.
