# Supplementary material for: Epidemiology, etiology, and diagnosis of health care acquired pneumonia including ventilator-associated pneumonia in Nepal
Source: PLoS One. 2021 Nov 17;16(11):e0259634. doi: 10.1371/journal.pone.0259634 (PMC8598067; doi:10.1371/journal.pone.0259634)
Supplement: Attachment — Submitted filename: Response to Reviewers.docx [file pone.0259634.s003.docx]

Journal Requirements:

When submitting your revision, we need you to address these additional requirements.

1. Please ensure that your manuscript meets PLOS ONE's style requirements, including those for file naming. The PLOS ONE style templates can be found at

https://journals.plos.org/plosone/s/file?id=wjVg/PLOSOne_formatting_sample_main_body.pdf and

<https://journals.plos.org/plosone/s/file?id=ba62/PLOSOne_formatting_sample_title_authors_affiliations.pdf>

Answer: Correction made

2. In your ethics statement, please specify the date of your ethics approval.

Answer: Date added : NHRC approval date: 11March 2016 and OxTREC approval date was 19 Oct 2018. The study was started after obtaining the approval from Nepal health Research Council (NHRC).

3. In your Data Availability statement, you have not specified where the minimal data set underlying the results described in your manuscript can be found. PLOS defines a study's minimal data set as the underlying data used to reach the conclusions drawn in the manuscript and any additional data required to replicate the reported study findings in their entirety. All PLOS journals require that the minimal data set be made fully available. For more information about our data policy, please see http://journals.plos.org/plosone/s/data-availability.

"Upon re-submitting your revised manuscript, please upload your study’s minimal underlying data set as either Supporting Information files or to a stable, public repository and include the relevant URLs, DOIs, or accession numbers within your revised cover letter. For a list of acceptable repositories, please see http://journals.plos.org/plosone/s/data-availability#loc-recommended-repositories. Any potentially identifying patient information must be fully anonymized.

Important: If there are ethical or legal restrictions to sharing your data publicly, please explain these restrictions in detail. Please see our guidelines for more information on what we consider unacceptable restrictions to publicly sharing data: http://journals.plos.org/plosone/s/data-availability#loc-unacceptable-data-access-restrictions. Note that it is not acceptable for the authors to be the sole named individuals responsible for ensuring data access.

We will update your Data Availability statement to reflect the information you provide in your cover letter.

[Note: HTML markup is below. Please do not edit.]

Answer: Dataset is submitted as S-Dataset_xlsx. relevant URLs, DOIs, or accession numbers within your revised cover letter.

Reviewers' comments:

Reviewer's Responses to Questions

Comments to the Author

1. Is the manuscript technically sound, and do the data support the conclusions?

The manuscript must describe a technically sound piece of scientific research with data that supports the conclusions. Experiments must have been conducted rigorously, with appropriate controls, replication, and sample sizes. The conclusions must be drawn appropriately based on the data presented.

Reviewer #1: Yes

Reviewer #2: Yes

________________________________________

2. Has the statistical analysis been performed appropriately and rigorously?

Reviewer #1: Yes

Reviewer #2: Yes

________________________________________

3. Have the authors made all data underlying the findings in their manuscript fully available?

The PLOS Data policy requires authors to make all data underlying the findings described in their manuscript fully available without restriction, with rare exception (please refer to the Data Availability Statement in the manuscript PDF file). The data should be provided as part of the manuscript or its supporting information, or deposited to a public repository. For example, in addition to summary statistics, the data points behind means, medians and variance measures should be available. If there are restrictions on publicly sharing data—e.g. participant privacy or use of data from a third party—those must be specified.

Reviewer #1: Yes

Reviewer #2: Yes

________________________________________

4. Is the manuscript presented in an intelligible fashion and written in standard English?

PLOS ONE does not copyedit accepted manuscripts, so the language in submitted articles must be clear, correct, and unambiguous. Any typographical or grammatical errors should be corrected at revision, so please note any specific errors here.

Reviewer #1: No

Reviewer #2: No

________________________________________

5. Review Comments to the Author

Please use the space provided to explain your answers to the questions above. You may also include additional comments for the author, including concerns about dual publication, research ethics, or publication ethics. (Please upload your review as an attachment if it exceeds 20,000 characters)

Reviewer #1: All abbreviations in the text must be spelled correctly (first in full).

English grammar needs to be revised.

CFU / ml must be spelled correctly in the text.

Answer: Corrected on the line 150 CFU/ml. The full form of CFU has been added to abbreviation list.

The sample was collected between 2016-2018, but the methods used CLSI 2016, this is a problem.

Answer: we corrected it to 2018. We checked the interpretation of the antibiotics that we tested against 2016 and 2018 CLSI guidelines and confirm that both have the same interpretation criteria for the antibiotics that we tested in this study.

What is the meaning of CONS(coagulase-negative staphylococci)? Please refer to it in Table 5.

Answer: CONS has been corrected as CoNS. The full form is coagulase negative *Staphyloccus*. Full form is added in the table 5.

In the first paragraph of the discussion, it is better to first explain about health care acquired pneumonia.

Answer: The data on HAP from prospective studies are scant notably from LMIC setting. This is added in the discussion

Reviewer #2: The study is very interesting ; results and conclusion are very useful mainly in this part of world with low resources setting. Authors have clearly discussed pneumonia among patients in ICU; compared according to intervention done. Simliarly, VAP incidence, mortality, etiological agents , Antimicrobial susceptibility tests and other clinical profiles were the items thoroughly discussed in the study. The author also suggests different control measures and preventive strategies. Overall, the paper is good quality but some shortcomings need to be addressed

In abstract

Only add about the main aim of study not all and present in the results serially.

Answer: We included only the main aim of the study in the abstract. This was followed by the summary of the statistical analysis.

Introduction

The authors have put excellent effort to justify the rationale and background behind the problem.

Methods

The authors have done well in describing methodology under different headings. It would be great if you could further clarify inclusion and exclusion criteria.

Answer: There were no exclusion criteria per se. One line was added in the study structure section: Patients who denied consent and patients under the age of 18 years of age were not included in the study.

Results

The authors have documented the results well mainly for association of intubation and pneumonia.

Discussion

It is good that you have tried to compare data of Nepal mainly VAP density with other Asian countries and LMICs. It would be nice to elaborate causes for contrasting results among these countries??

Answer: In high-income countries, a combination of surveillance, education, and tailored intervention and prevention bundles have led to a reduction in the incidence of VAP

Why contrasting findings were seen among your study and other western countries study. What might be the likely cause for low numbers of gram positive organism?

Answer: We are unable to provide explanation based on the findings from the study. There could be multidimensional factors ranging from patient care management protocols, antibiotic stewardship program, VAP bundle implementation, and continuous training and surveillance system.

As Nepal is poor in economy, please add some cost-effective control and preventive measures of HAP and VAP.

Answer: Guidelines for VAP prevention, including hand washing, elevation of the head of the bed, oral care with chlorhexidine, optimized endotracheal tube cuff pressure, respiratory circuit manipulation, and weaning protocols to early extubation were established in our hospital. These are cost effective control and preventive measures of VAP. Strict compliance, staff training, and regular monitoring of implementation of such guidelines will be effective in the prevention of VAP. This is added in the discussion

As the outcomes are relevant , possible description of risk factors of VAP in country like Nepal can also be included.

Answer: We have analysed data by grouping them into categories of intubation required and non intubated group and further divided into five categories into VAP development. We measured various variables and their association to each group.

Are ventilator care bundles used in your study site?

Answer: Guidelines for VAP prevention, including hand washing, elevation of the head of the bed, oral care with chlorhexidine, optimized endotracheal tube cuff pressure, respiratory circuit manipulation, and weaning protocols to early extubation were established in our hospital. These are cost effective control and preventive measures of VAP. Strict compliance, staff training, and regular monitoring of implementation of such guidelines will be effective in the prevention of VAP.

Please add any strengths and limitations above conclusion in your study if any.

Answer: These lines in the conclusion section explains the strength of this study.

Pneumonia was one of the common infections in our ICU setting. Pneumonia developed among intubated patients in the ICU had longer days of stay in the ICU when compared to non-intubated patients with pneumonia. We found high VAP incidence in this study and highest mortality was observed among VAP patients followed by non VAP (I+P+V-) patients. MDR Gram negative bacteria were the dominant organisms from both VAP and non-VAP categories.

One of the limitations of this study was that it was conducted at a single hospital. Surveillance studies on HAIs at various hospitals within the country are required in identifying the etiological agents. Antimicrobial susceptibility profiles of the etiological agents and outcomes of HAP and VAP would be beneficial for setting more appropriate guidelines for management of such diseases.

Minor issues

Spacing not done in some places like line no. 58

Answer: Corrected

Referencing no. Should be after full-stop.

Answer: we referred to the Plos one formatting style.

In table 1, write unit of weight , age

Answer: Corrections were made. Added kilogram as a unit of weight and Years along with age in table 1.

Page 266 to 230 n"=" is missing

And also Page 277 to 280 ; and other lines.

Answer: Corrected throughout the manuscript.

Brackets are only open or only closed some where.

Answer: Checked throughout the manuscript and corrected

Table 4 and 5 can be merged into a single table.

Answer: We have removed table 4 as this information is depicted in Fig 5. We are keeping Table 5 as it is (without merging it to table 4).

Correct typographical errors.

Answer: Done

Thank you!

________________________________________

6. PLOS authors have the option to publish the peer review history of their article (what does this mean?). If published, this will include your full peer review and any attached files.

If you choose “no”, your identity will remain anonymous but your review may still be made public.

Do you want your identity to be public for this peer review? For information about this choice, including consent withdrawal, please see our Privacy Policy.

Reviewer #1: No

Reviewer #2: No

[NOTE: If reviewer comments were submitted as an attachment file, they will be attached to this email and accessible via the submission site. Please log into your account, locate the manuscript record, and check for the action link "View Attachments". If this link does not appear, there are no attachment files.]

While revising your submission, please upload your figure files to the Preflight Analysis and Conversion Engine (PACE) digital diagnostic tool, https://pacev2.apexcovantage.com/. PACE helps ensure that figures meet PLOS requirements. To use PACE, you must first register as a user. Registration is free. Then, login and navigate to the UPLOAD tab, where you will find detailed instructions on how to use the tool. If you encounter any issues or have any questions when using PACE, please email PLOS at figures@plos.org. Please note that Supporting Information files do not need this step.
